# Accuracy and generalizability of using automated methods for identifying adverse events from electronic health record data: a validation study protocol

**DOI:** 10.1186/s12913-017-2069-7

**Published:** 2017-02-16

**Authors:** Christian M. Rochefort, David L. Buckeridge, Andréanne Tanguay, Alain Biron, Frédérick D’Aragon, Shengrui Wang, Benoit Gallix, Louis Valiquette, Li-Anne Audet, Todd C. Lee, Dev Jayaraman, Bruno Petrucci, Patricia Lefebvre

**Affiliations:** 1 School of Nursing, Faculty of Medicine and Health Sciences, University of Sherbrooke, 3001, 12e Avenue Nord, Sherbrooke, QC J1H 5N4 Canada; 2 Centre de recherche de l’Hôpital Charles-LeMoyne, University of Sherbrooke—Campus Longueuil, 150 Place Charles-LeMoyne, Longueuil, QC J4K 0A8 Canada; 3 Department of Epidemiology, Biostatics and Occupational Health, Faculty of Medicine, McGill University, Purvis Hall, 1020 Pine Avenue West, Montreal, QC H3A 1A2 Canada; 4 Department of Quality, Patient Safety and Performance, McGill University Health Centre, 2155 Guy Street, Montreal, QC H3H 2R9 Canada; 5 Ingram School of Nursing, McGill University, Wilson Hall, 3506 University Street, Montreal, QC H3A 2A7 Canada; 6 Department of Anesthesiology, Faculty of Medicine and Health Sciences, University of Sherbrooke and Centre hospitalier universitaire de Sherbrooke, 3001, 12e Avenue Nord, Sherbrooke, QC J1H 5N4 Canada; 7 Faculty of Sciences, Department of Informatics, University of Sherbrooke, 2500 Boulevard de l’Université, Sherbrooke, QC J1K 2R1 Canada; 8 Department of Diagnostic Radiology, McGill University and McGill University Health Centre, 1650 Cedar Avenue, Montreal, QC H3G 1A4 Canada; 9 Department of Microbiology and Infectious Diseases, University of Sherbrooke and Centre hospitalier universitaire de Sherbrooke, 3001, 12e Avenue Nord, Sherbrooke, QC J1H 5N4 Canada; 10 Department of Internal Medicine, McGill University and McGill University Health Centre, 1650 Cedar Avenue, Montreal, QC H3G 1A4 Canada; 11 Department of Quality, Evaluation, Performance and Ethics, Centre hospitalier universitaire de Sherbrooke, 3001, 12e Avenue Nord, Sherbrooke, QC J1H 5N4 Canada

**Keywords:** Adverse events, Electronic health record, Acute care hospital, Automated detection, Natural language processing, Patient safety, Data warehouse

## Abstract

**Background:**

Adverse events (AEs) in acute care hospitals are frequent and associated with significant morbidity, mortality, and costs. Measuring AEs is necessary for quality improvement and benchmarking purposes, but current detection methods lack in accuracy, efficiency, and generalizability. The growing availability of electronic health records (EHR) and the development of natural language processing techniques for encoding narrative data offer an opportunity to develop potentially better methods. The purpose of this study is to determine the accuracy and generalizability of using automated methods for detecting three high-incidence and high-impact AEs from EHR data: a) hospital-acquired pneumonia, b) ventilator-associated event and, c) central line-associated bloodstream infection.

**Methods:**

This validation study will be conducted among medical, surgical and ICU patients admitted between 2013 and 2016 to the *Centre hospitalier universitaire de Sherbrooke* (CHUS) and the McGill University Health Centre (MUHC), which has both French and English sites. A random 60% sample of CHUS patients will be used for model development purposes (cohort 1, development set). Using a random sample of these patients, a reference standard assessment of their medical chart will be performed. Multivariate logistic regression and the area under the curve (AUC) will be employed to iteratively develop and optimize three automated AE detection models (i.e., one per AE of interest) using EHR data from the CHUS. These models will then be validated on a random sample of the remaining 40% of CHUS patients (cohort 1, internal validation set) using chart review to assess accuracy. The most accurate models developed and validated at the CHUS will then be applied to EHR data from a random sample of patients admitted to the MUHC French site (cohort 2) and English site (cohort 3)—a critical requirement given the use of narrative data –, and accuracy will be assessed using chart review. Generalizability will be determined by comparing AUCs from cohorts 2 and 3 to those from cohort 1.

**Discussion:**

This study will likely produce more accurate and efficient measures of AEs. These measures could be used to assess the incidence rates of AEs, evaluate the success of preventive interventions, or benchmark performance across hospitals.

## Background

Adverse events (AEs) are injuries caused by medical management rather than by the underlying condition of the patient [1]. AEs in acute care hospitals are frequent and associated with significant morbidity, mortality and costs [2, 3]. For this reason, preventing AEs is a high priority worldwide [4, 5]. To evaluate the success of preventive measures, there is a need for accurate, timely and efficient methods for monitoring AE rates [6, 7]. Moreover, with the growing emphasis on benchmarking and public reporting of AE data, these methods must allow for valid inter-institutional comparisons [8, 9]. However, at present, there are no such methods.

Indeed, hospitals typically rely on manual chart review, prevalence surveys, incident reporting systems or discharge diagnostic codes for monitoring AE rates [10]. Manual chart review is a time-consuming, resource-intensive and costly process [6, 11]. As a consequence, it is an impractical means for the routine detection and hospital-wide monitoring of AEs. Prevalence surveys similarly lack in efficiency and scalability and are subject to important inter-observer variations in the reported AE rates [11, 12]. Incident reporting systems are known to significantly underestimate the true incidence rate of AEs [13]. Discharge diagnostic codes have low sensitivity and positive predictive value (PPV) for detecting AEs [14]. Moreover, important variations in coding practices across institutions preclude their use for benchmarking purposes [14, 15]. Thus, the limitations in existing methods for measuring AEs have curtailed the ability to conduct continuous quality monitoring in acute care hospitals and the capacity to benchmark performances across institutions.

With the advent of electronic health records (EHR), and the development of automated methods for encoding and classifying EHR data, an exciting opportunity has emerged to develop potentially better methods of AE detection. Moreover, with the growing adoption of standards for storing and exchanging EHR data across applications and institutions [16], there is an opportunity to develop methods of AE detection that are potentially generalizable; a key requirement to valid benchmarking.

Taking advantage of these new opportunities, researchers have started to develop novel and potentially more accurate and efficient methods of AE detection, such as the natural language processing (NLP) of clinical narratives [12, 17, 18]. For instance, in 2012, we received funding from the Canadian Institutes of Health Research (CIHR) to examine the accuracy of NLP techniques for identifying venous thromboembolism (VTE) from electronic narrative radiology reports. We found that NLP techniques are highly efficient and accurate in identifying this AE [19, 20]. While VTEs can be objectively detected from a single source of EHR data (i.e., narrative radiology reports), this is not the case for most AEs (e.g., pneumonia). For these events, several sources of EHR data must be combined to satisfy existing case definitions (e.g. microbiology, laboratory, radiology, vital signs) [21–23]. However, the accuracy and generalizability of such AE detection models are unknown [6, 18].

To move the field forward, there is a strong need to determine the accuracy of AE detection models that integrate the information from all available EHR data sources [6]. Moreover, to obtain valid interinstitutional comparisons, the generalizability of these models to other acute care hospitals, including both French and English settings—which is essential given their reliance on narrative data,—must be established. The proposed study aims to address these requirements.

Specifically, this study aims to determine the accuracy and generalizability of using automated methods for detecting AEs from EHR data. Three AEs were selected for the purpose of this study: a) hospital-acquired pneumonia, b) ventilator-associated events and, c) central line-associated bloodstream infection. The rationale for selecting these AEs is provided in the Methods section.

## Methods

### Settings

This study will be conducted at two leading Canadian academic health centres: 1) Centre hospitalier universitaire de Sherbrooke (CHUS) and, 2) McGill University Health Centre (MUHC). The CHUS is composed of two acute care hospitals and has close to 700 beds. It serves a population of 500,000 people with annual volumes of 32,000 hospitalizations, 27,000 surgical procedures and 4500 intensive care unit (ICU) admissions [24]. The MUHC is composed of three acute care hospitals, including a French site and two English sites, and has more than 800 adult beds. It serves a population of 1.7 million people, with annual volumes of 40,000 hospitalizations, 33,300 surgical procedures and 6000 ICU admissions [25].

### Design and population

**Fig. 1:**
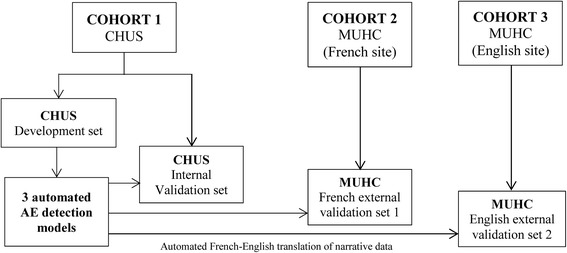
Project design

The study population consists of all adult medical, surgical and ICU patients admitted to the CHUS and the MUHC between January 1, 2013 and December 31, 2016. Our proposed approach to AE detection model development and validation builds on and expand our prior research work in the area [26]. First, we will use a random 60% sample of all patients admitted to the CHUS between the aforementioned dates for model development purposes (cohort 1, development set) (Fig. 1). Then, using a random sample of these patients, a reference standard assessment of their medical chart will be performed to determine their true AE status (i.e., positive or negative). Using the manually reviewed cases as the reference standard, three automated AE detection models will be iteratively developed and optimized (i.e., one for each AE of interest, which are hospital-acquired pneumonia [HAP], ventilator-associated events [VAE], and central line-associated bloodstream infection [CLABSI]). These models will be developed to mirror published AE definitions (e.g., Centers for Disease Control and Prevention/National Healthcare Safety Network [CDC/NHSN] surveillance definitions) [21–23], which will also guide electronic health record (EHR) data extraction at the CHUS (Table 1). The most accurate models will then be validated on a random sample of the remaining 40% of CHUS patients (cohort 1, internal validation set), and a reference standard assessment of the medical chart will be performed (Fig. 1) [26].

**Table 1:** Data sources and CDC/NHSN criteria for determining adverse event (AE) occurrence

HOSPITAL-ACQUIRED PNEUMONIA (HAP)				
Radiology	Microbiology	Laboratory	Vital signs	Pharmacy
- ≥ 1 chest imaging test results with pneumonia-related findings (e.g., air-space disease, consolidation, infiltrate, focal opacification).	- Gram stain of sputum or pleural fluid sample with ≥ 25 neutrophils and ≤ 10 squamous epithelial cells per low power field x100.	- WBC ≤ 4000/mm^3^ or; - WBC ≥ 12,000/mm^3^ or; - Abnormal trends in WBC for ≥ 48 h.	- Temperature > 38°C or abnormal trends in temperature ≥ 48 h; - RR > 25 breaths/min.; - O_2_ desaturations < 94%; - HR > 100 beats/min	- A HAP-related antimicrobial agent^a^ is started, and is continued for ≥ 4 days.
VENTILATOR-ASSOCIATED EVENT (VAE)				
Ventilator settings	Microbiology	Laboratory	Vital signs	Pharmacy
- ↑ daily min. FiO_2_ of ≥ 0.2 point or ↑ daily min. PEEP values of ≥ 3cmH_2_O sustained for ≥ 48 h, as compared to a baseline period of ≥ 48 h of stability or improvements on the ventilator in terms of FiO_2_ or PEEP values.	- Culture meeting one of the following thresholds and growing a recognized pathogen^b^: a) EA: ≥ 10^5^ CFU/ml b) BAL: ≥ 10^4^ CFU/ml c) LT: ≥ 10^4^ CFU/g; d) PSB: ≥ 10^3^ CFU/ml e) Gram stain of resp. secretions (see: HAP)	- WBC ≤ 4000/mm^3^ or; - WBC ≥ 12,000/mm^3^ or; - Abnormal trends in WBC for ≥ 48 h.	- Temperature > 38°C or <36°C for 48 h, or abnormal trends in temperature ≥ 48 h.	- A new antimicrobial agent^a^ is started, and is continued for ≥ 4 days.
			ICU Database	Radiology
			- Presence of an endotracheal tube and of mechanical ventilation	- For descriptive purposes, whether chest imaging test results provide evidence of pneumonia, pulmonary edema, atelectasis or ARDS.
CENTRAL LINE-ASSOCIATED BLOODSTREAM INFECTION (CLABSI)				
Radiology	Microbiology	Laboratory	Vital signs	Pharmacy
- Chest imaging test results with evidence of at least one central line in place.	- Recognized pathogen(s)^b^ identified from ≥ 1 blood specimens by a culture based micro-biologic testing method, with the organism identified in blood not related to an infection at another site.	- WBC ≤ 4000/mm^3^ or; - WBC ≥ 12,000/mm^3^ or; - Abnormal trends in WBC for ≥ 48 h.	- Temperature > 38°C or; <36°C for ≥ 48 h or; - Abnormal trends in temperatures for ≥ 48 h; -hypotension with systolic pressure ≤ 90 mmHg.	- A CLABSI-related antimicrobial agent^a^ is started, and is continued for ≥ 4 days.

*Abbreviations*: *ARDS* acute respiratory distress syndrome, *BAL* brochoalveolar lavage, *BSI* bloodstream infection, *CFU* colony forming units, *EA* endotracheal aspirate, *FiO*_*2*_ Fraction of inspired oxygen, *HR* heart rate, *LT* lung tissue, *PEEP* positive end-expiratory pressure, *PSB* protected specimen brush, *RR* respiratory rate, *WBC* white blood cell count

^a^ The eligible antimicrobial agents are listed in the CDC/NHSN guidelines

^b^ The list of recognized pathogens is defined in the CDC/NHSN guidelines

To determine the extent to which these models can be generalized to other acute care settings (including both French and English hospitals)—a critical requirement given the reliance of these models on narrative data—, the most accurate models developed and validated at the CHUS will be applied to a random sample of patients admitted to the MUHC French site (cohort 2, French external validation set) and to the MUHC English sites (cohort 3, English external validation set), and a reference standard assessment of the medical chart will be performed. Prior to applying the models to data from the MUHC English sites, French narrative data employed as predictor of AE occurrence in the CHUS models will be translated into English using a previously validated natural language processing (NLP) approach [27].

### Data sources

Data required for developing the AE detection models will be extracted from the CHUS and the MUHC information systems and clinical data warehouses, and will be linked by unit, patient, and hospital admission date. Specifically, data will be extracted from eight electronic databases: 1) radiology, 2) laboratory, 3) microbiology, 4) pharmacy, 5) vital signs, 6) admission, discharge, and transfer, 7) intensive care unit, and 8) hospital discharge abstracts (Table 1). Narrative data from these sources (e.g., radiology reports) will be converted to numeric using NLP techniques developed and validated in our prior research work [19, 20].

### Measures

#### Adverse events

Three potentially preventable AEs were selected for the purpose of this study: a) hospital-acquired pneumonia (HAP); defined as an infection of the lung parenchyma occurring 48 h or more after hospital admission [21], b) ventilator-associated event (VAE); an AE indicator that was introduced by the CDC in January 2013 to broaden the focus of surveillance in ventilated patients from pneumonia alone to a larger set of physiologically significant and potentially preventable complications of mechanical ventilation, including pulmonary edema, acute respiratory distress syndrome, and/or atelectasis [28], and, c) central line-associated bloodstream infection (CLABSI) defined as a laboratory-confirmed bloodstream infection occurring in a patient with a central line in place for more than 48 h on the date that the positive blood culture is identified [23].

These AEs were selected because they are associated with significant morbidity, mortality, and costs [29–31]. Moreover, these indicators have high incidence rates compared to other AEs. HAP accounts for 15% of all hospital-acquired infections and 25% of all ICU-acquired infections [30]. HAP is estimated to occur at a rate of 5 to 20 cases per 1000 hospital admissions [30]. VAEs are the most frequent ICU-acquired AEs; occurring in 5.6% to 10% of mechanically ventilated patients [31]. Lastly, central lines are the most important cause of bloodstream infections, with CLABSIs occurring in 2% to 7% of all catheterizations [32].

#### Patient demographic and clinical characteristics

Patient demographic characteristics, comorbidities and severity of illness can influence the likelihood of AE occurrence, the accuracy of AE detection models and the generalizability of these models across institutions [11]. Patient age and sex will be extracted from the discharge abstract database. Comorbidities will be measured using the Charlson Comorbidity Index, a weighted index of 17 comorbidities [33]. Comorbidities will be measured at the time of hospital admission using discharge diagnostic codes from prior hospitalizations since 2008 (i.e., the earliest date for which complete data is available at the study sites). Severity of illness in medical and surgical patients will be measured within 24 h of hospital admission using the Laboratory-based Acute Physiology Score (LAPS); a scoring system that integrates information from 14 laboratory tests into a single continuous variable [34]. Severity of illness in ICU patients will be measured using the Acute Physiology and Chronic Health Evaluation (APACHE) III Score; a scoring system that integrates 12 physiologic measurements [35]. APACHE III scores are systematically measured at the CHUS and the MUHC within 24 h of ICU admission and stored in the ICU database.

#### Reference standard development and validation

Charts will be reviewed by trained medical chart reviewers (MCRs) who will perform chart review using standardized surveillance definitions (i.e., CDC/NHSN) [21–23]. MCRs will enter patient AE status (i.e., positive or negative) in an electronic abstraction form that was developed during our pilot work [19, 20]. To assess inter-rater reliability, a random 10% sample of the medical charts will be blindly reviewed by a second MCR, and intraclass correlation coefficients (ICC) will be computed. ICC values above 0.75 will be judged as excellent [36]. To ensure data quality throughout the study, MCRs will undergo periodic quality assurance monitoring [26].

#### AE detection model development and optimization

The automated AE detection models will be developed in accordance with published methodological guidelines [37, 38], and will mirror CDC/NHSN surveillance definitions (Table 1) [21–23]. Three successive steps will be followed, which build on and expand on our prior research work in the area [26]. In Step 1, receiver operating characteristic (ROC) curves will be used to determine for selected EHR data sources: a) optimal cut points for defining the presence of an AE (e.g., using various thresholds for defining an elevated white blood cell count, an abnormal ventilator setting or an elevated body temperature), and b) optimal time window for measuring these parameters (e.g., requiring a single day with an elevated white blood cell count versus two or more consecutive days, requiring only one versus two or more consecutive chest x-rays showing evidence of pneumonia) (Table 1) [26]. In addition, ROC area under the curve (AUC), along with its 95% confidence intervals (CIs), will be used to assess the accuracy of each individual data source. To analyse narrative data, we will build on NLP techniques developed in our prior research work to identify subsets of words, phrases and patterns in clinical narratives that are significantly associated with the occurrence of each AE of interest [19, 20]. In Step 2, three separate multivariate logistic regression analyses—one for each AE of interest—will be conducted to assess the incremental effect in detection accuracy of combining EHR data sources, using the optimal cut points and measurement windows identified in Step 1 [26]. Stepwise and backward procedures will be used to identify data sources that are significantly associated with AE occurrence [37]. AUCs along with their 95%CIs will be used to assess the incremental effect in detection accuracy associated with the inclusion of a given data source in the regression model. AUCs across models will be compared [38]. Data sources not significantly associated with AE occurrence will be eliminated from the model [26]. In Step 3, the best regression models identified in Step 2 will be used to assess the incremental effect in detection accuracy of including patient demographic characteristics, comorbidities and severity of illness [26]. Specifically, the AUCs of regression models including these characteristics will be compared to those from the best performing models in Step 2 [38]. During each of the aforementioned steps, estimates of sensitivity, specificity, positive predictive value (PPV) and negative predictive value (NPV), along with their 95% CIs, will be computed [26].

#### AE detection model validation and update

The best performing models from the development and optimization steps will be applied to a random sample of the remaining 40% of CHUS patients (cohort 1, internal validation set) and their performance will be assessed using a reference standard assessment of the medical chart. AUCs from the validation set will be compared to those obtained during the development and optimization steps [38]. Estimates of sensitivity, specificity, PPV and NPV, along with their 95% CIs will be computed for the best performing models.

To assess the extent to which the best performing models developed and validated at the CHUS can be generalized to other acute care hospitals, they will be applied to a cohort of patients admitted to the MUHC French site (cohort 2, French validation set) as well as to a cohort of patients admitted to the MUHC English sites (cohort 3, English validation set) (Fig. 1). Then, a reference standard assessment of the medical charts will be performed at each site for a random sample of AE positive and AE negative patients. Prior to applying the models to data from the MUHC English site, French words used as predictors of AE occurrence in the CHUS models will be translated into the equivalent English terms using a validated NLP approach [27]. To determine if there are any significant differences in the performance of the AE detection models across sites, the AUCs obtained from cohort 2 and 3 will be compared to those obtained from the best performing models in cohort 1. Lastly, because it is common for the performances of prediction models to degrade when validated in a new patient population, the intercept and the regression coefficients of the CHUS models will be recalibrated (updated), if necessary, on MUHC data [37].

#### Sample size requirements

For the development set, assuming an incidence rate of 5.0% for both HAP and CLABSI [30, 32], and of 7% for VAE [31] a total of 894 AE positive charts (i.e., 298 HAP, 298 VAE and 298 CLABSI) and 5662 AE negative charts is required to generate a 95%CI width of 0.10 around a sensitivity estimate of 0.90 [39]. For the validation sets, we will maximize efficiency by using the automated AE detection models to oversample AE positive patients in relation to AE negative ones [40]. Assuming the aforementioned incidence rates, a total of 639 AE positive (i.e., 237 HAP, 165 VAE and 237 CLABASI) and 3099 AE negative charts is required in each validation set to generate a 95%CI width of 0.10 around a sensitivity estimate of 0.90 that is adjusted for the over-sampling of AE positive patients [40]. To minimize the costs associated with performing chart review, all AE negative patients in the validation sets will be selected so that they are negative for all three AEs according to the AE detection models.

## Discussion

### Current study status

**Fig. 2:**
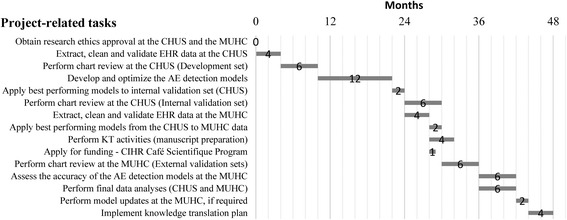
Gantt chart for project timelines

This study was funded by the CIHR in July 2016. We received research ethics approval from the CHUS and the MUHC in August 2016 and are now ready to initiate data extraction at the CHUS. This study will be conducted over 4 years. The details of the study timelines are provided in Fig. 2.

### The anticipated contributions

This study aims to produce more accurate and efficient measures of AEs. These measures could be used to document the incidence rates of AEs, evaluate the effectiveness of interventions targeted at improving patient safety and monitor progresses through time. In addition, because these measures are automatable, they offer the potential to rapidly scan high volumes of EHR data with minimal human input and at relatively low costs, which represent major gains compared to using manual chart review or prevalence surveys. As a result, human resources currently assigned to AE surveillance in acute care hospitals could be more productively reallocated to the development and implementation of preventive interventions. Lastly, automation has the potential to standardize AE surveillance; a net gain over manual approaches and a critical requirement to valid interinstitutional comparisons. Such comparisons are needed to define targets for performance improvement, but also to identify and implement best practices from leaders in the field.

### Potential challenges and mitigation strategies

Based on our prior research work at the CHUS and the MUHC, we anticipate three potential challenges. First, EHR data extraction is often delayed by conflicting priorities. To mitigate this challenge, and ensure that the study is conducted within the proposed timelines, we: a) are working on AE indicators that are highly relevant to the CHUS and the MUHC, b) have invited key decision-makers from these institutions as co-investigators/collaborators on this study. These decision-makers have authority over the data warehouses at the CHUS and MUCH; the main infrastructure required for supporting the proposed study. They are also important knowledge and technology users; bringing practice-relevant knowledge to the team. Second, while infection preventionists (IPs) routinely monitor HAP, VAE and CLABSI rates, existing data at the study sites is only available for small subsets of selected patients and time periods (as in most other hospitals). Moreover, important variations in the application of surveillance definitions by IPs both within and across hospitals preclude the use of this data as a reference standard [7]. For these reasons, we opted to develop and validate our own reference standard for this study. Last, the performances of prediction models often degrade when they are validated in a new patient population. To guard against this, and maximize the generalizability of the AE detection models, we have planned for model update techniques in the data analysis steps.

### Knowledge translation plan

To facilitate the dissemination and uptake of the new knowledge that will be generated by this study, our knowledge translation plan will target four groups. First, we have partnered with key decision-makers, clinical leaders and patient safety experts at the CHUS and the MUHC who are engaged as co-investigators/collaborators on the study, have significantly contributed to its development and to the selection of high-priority AE indicators. Through such engagement, we aim to develop practice-relevant and clinically useful AE detection models. Moreover, based on the results of our pilot work at these sites [19, 20], we are exploring the possibility of integrating the AE detection models within quality and safety dashboards at the CHUS and the MUHC. These could serve as demonstration projects for other hospitals throughout Canada and abroad. Second, to reach a national audience of potential knowledge users (i.e., patient safety experts, infection control professionals), we will partner with the Communication Services at the University of Sherbrooke to organize and advertise two national webinars (one in French, the other in English) through Mybys web-conferencing technologies (www.mybys.com). These webinars will be tailored to the needs of this audience and will aim to increase awareness about automated AE detection using EHR data, while highlighting key messages and lessons from our research study. Third, we will organize press conferences to inform the population and the media about the findings of this study and the value-added of EHRs for patient safety. Lastly, we will communicate the findings of this study to academic and research colleagues through conference presentations and submission of manuscripts for publication.

## Abbreviations

AE: Adverse event
APACHE: Acute Physiology and Chronic Health Evaluation
AUC: Area under the curve
CDC/NHSN: Centers for Disease Control and Prevention/National Healthcare Safety Network
CHUS: Centre hospitalier universitaire de Sherbrooke
CIHR: Canadian Institutes of Health Research
CLABSI: Central line-associated bloodstream infection
EHR: Electronic health record
HAP: Hospital-acquired pneumonia
ICC: Intraclass correlation coefficients
ICU: Intensive care unit
IP: Infection preventionist
LAPS: Laboratory-based Acute Physiology Score
MCR: Medical chart reviewers
MUHC: McGill University Health Centre
NLP: Natural language processing
NPV: Negative predictive value
PPV: Positive predictive value
ROC: Receiver operating characteristic curve
VAE: Ventilator-associated event
VTE: Venous thromboembolism
